# Effect of local zoledronate on implant osseointegration in a rat model

**DOI:** 10.1186/1471-2474-13-42

**Published:** 2012-03-22

**Authors:** David A Back, Stephan Pauly, Lisa Rommel, Norbert P Haas, Gerhard Schmidmaier, Britt Wildemann, Stefan H Greiner

**Affiliations:** 1 Department of Orthopedics and Traumatology, German Armed Forces Hospital Berlin, Berlin, Germany; 2 Center for Musculoskeletal Surgery, Charité-Universitaetsmedizin, Berlin, Germany; 3 Julius Wolff Institute and Berlin-Brandenburg Center for Regenerative Therapies, Charité-Universitaetsmedizin, Berlin, Germany; 4 Department for Orthopedics, Traumatology and Paraplegiology, University of Heidelberg, Heidelberg, Germany

## Abstract

**Background:**

An implant coating with poly(*D, L*-lactide) (PDLLA) releasing incorporated Zoledronic acid (ZOL) has already proven to positively effect osteoblasts, to inhibit osteoclasts and to accelerate fracture healing. Aim of this study was to investigate the release kinetics of the chosen coating and the effect of different concentrations of ZOL locally released from this coating on the osseointegration of implants.

**Methods:**

For release kinetics the release of C14-labled ZOL out of the coating was monitored over a period of six weeks in vitro. For testing the osseointegration, titanium Kirschner wires were implanted into the medullary canal of right femurs of 100 Sprague Dawley rats. The animals were divided into five groups receiving implants either uncoated or coated with PDLLA, PDLLA/ZOL low (1.2% w/w) or PDLLA/ZOL high (2% w/w). Additionally, a group with uncoated implants received ZOL intravenously (i.v.). After 56 days animals were sacrificed, femurs dissected and either strength of fixation or histological bone/implant contacts and newly formed bone around the implants were determined.

**Results:**

Release kinetics revealed an initial peak in the release of C14-ZOL with a slight further progression over the following weeks. There was no significant enhancement of osseointegration for both groups who received ZOL-coated implants or ZOL i.v. compared to the controls in biomechanical or histological analyses, except for a significant raise in strength of fixation of ZOL i.v. versus PDLLA.

**Conclusions:**

Even though the investigated local ZOL application did not enhance the osseointegration of the implant, the findings might support its application in fracture treatment, since fracture stabilization devices are often explanted after consolidation.

## Background

Implants commonly used in orthopedic surgery serve on the one hand as fixation devices after fractures, granting stabilization until fracture healing. On the other hand implants are used for joint replacement and a persistent osseointegration is desired. However, failure of osseointegration in joint replacement is a frequent complication. To address this issue, research has recently been focused on the supplementary use of drugs that are known to influence bone turnover. In particular, the class of Bisphosphonates (BPs) has been evaluated for improvement of both, fracture healing and osseointegration in animal models [1-8] and clinical studies [9,10].

BPs such as Zoledronic acid (ZOL) are in clinical use for prevention and treatment of skeletal diseases associated with increased bone resorption like malignant tumors [11,12] or osteoporosis [8,9]. As BPs have a strong affinity to Calcium, they almost exclusively concentrate in bone mineral, mainly in regions of bone resorption or formation. Incorporated by osteoclasts during bone resorption, Nitrogen-containing BPs such as ZOL block the farnesyl pyrophosphate synthase, a crucial enzyme in the mevalonate pathway, which interferes with the prenylation of proteins causing functional impairment of osteoclasts and thus a decrease in bone resorption [13].

The influence of BPs on bone healing and the osseointegration of implants have been widely investigated. During bone repair, BPs have been shown to have an anti-catabolic, almost net anabolic effect [14,15]. In most studies, this resulted in the enhancement of fracture consolidation [16,17]. Concerning osseointegration, studies focused on the effect of systemically or locally applied BPs on implant fixation [10,18]. Locally, BPs were either applied directly to the implant site or linked to the implant with a coating substance as drug carrier [1,19]. Different coatings were tested using especially calcium phosphates like hydroxyapatite [20-22] or cross-linked fibrinogen layer [23-25]. In most cases, application of BPs showed beneficial biomechanical and histological effects on implant osseointegration [1,23,25-27]. However, in some studies, BPs did not improve implant fixation or even worsened it [28-30].

Previous in vitro studies investigated the effect of locally released ZOL from a poly(*D, L*-lactide) (PDLLA) implant coating on human osteoblasts and osteoclasts. A dose dependent promoting effect on osteoblasts [31] and a decrease in osteoclast formation and osteoclastic resorption activity [32] could be shown. In a coculture of human osteoblasts and osteoclasts, beneficial effects on osteoblast differentiation and protein synthesis and a decrease of osteoclast formation with no significant decrease in osteoclastic resorption activity has also been shown [33]. Moreover, in a rat fracture model, local application of ZOL delivered from an intramedullary implant showed a significant increase in biomechanical strength of the fracture side [17].

Aims of the study were first to investigate the release kinetics of the chosen PDLLA/ZOL coating. Second, to analyze the effect of the coating on osseointegration of implants using an established non-weight-bearing rat implant model [34]. Third, to detect possible differences of the local application of ZOL in comparison to systemic application.

## Methods

### Release kinetics

To detect the release profile of ZOL from PDLLA, an in vitro elution experiment was performed. Titanium K-wires were dip coated in a solution with Carbon 14(C14)-labeled ZOL (Novartis Pharma AG, Basel, Switzerland) dissolved in PDLLA/ethyl acetate resulting in an estimated concentration of 50 μg per sample. Ten Titanium K-wires with PDLLA/C14-ZOL coating were sterile incubated at 37°C (each in 5.2 mL 0.9% NaCl). At time points 0 min, 10 min, 1 h, 6 h, 12 h, 24 h, 48 h, 4 d, 7 d, 14 d, 28 d, and 42 d 100 μL were drawn from each solution. The concentrations of C14-ZOL were detected indirectly via radioactivity count as automatically quench-corrected Counts Per Minute (CPM) by a Liquid Scintillation Counter (Wallac Oy, Turku, Finland).

### Coating of the implants and groups

Coating of the implants was performed as described previously [35]. After dissolving 100 mg PDLLA in 1.5 ml ethyl acetate at room temperature, the solution was sterile filtered. ZOL was dissolved in ethyl acetate/PDLLA solution to obtain a concentration of 1.2% w/w or 2% w/w. Sterile Kirschner wires (1.4-mm diameter, Synthes^®^, Paoli, USA) were used as implants for the experiments. They were dipped two times into the coating solution and dried under laminar air flow conditions. For systemic application ZOL was dissolved in NaCl to obtain a concentration of 0.1 mg/ml. The study contained five groups:

- - Group I (Control)-uncoated K-wire

- - Group II (PDLLA)-PDLLA

- - Group III (ZOL low)-PDLLA + ZOL low dose (20 μg, 1.2% w/w)

- - Group IV (ZOL high)-PDLLA + ZOL high dose (50 μg, 2% w/w)

- - Group V (ZOL i.v.)-uncoated + ZOL i.v. 0.1 mg/kg

Zoledronic acid as a pure substance was kindly provided by Novartis Pharma AG (Basel, Switzerland).

### Animals and experimental design

Animals used for experiment were 100 five-month-old female Sprague Dawley rats (Harlan-Winkelmann, Germany) with a mean body weight of 267 g +/- 25 g (SD). They were kept in groups of five rats each. For anesthesia isoflurane gas (Forene^®^) and an intraperitoneal injection with a mixture of Ketaminhydrochlorid (100 mg/ml per 80 mg/kg body weight) and Xylazin 2% (12 mg/kg body weight) were used. The right hind leg was shaved for operation. A 3 mm longitudinal incision was made to split the skin and the patellar ligament. Using a 1.2 mm hand drill, the intracondylar notch of the right femur was reamed. Thereafter, insertion of the implant into the medullary cavity to the proximal end was performed in a retrograde manner. Radiographs were taken to verify the correct position of the implant using the trochanter major as control point. At the end of operation the extending part of the implant was cut off and skin wounds were sutured. For systemic application of ZOL either the cephalic vein or the left greater saphenous vein were used. Immediately after implant insertion, ZOL dissolved in 1 ml NaCl was injected into the vein.

After the operation and on the first postoperative day all animals received subcutaneous injections of buprenorphine (Temgesic^®^, 0.05 mg/kg) for analgesia. Using a precision scale, body weight was determined and body temperature was measured rectally at days 0 (OP) and 56. Wound healing and signs of a local or systemic infection were evaluated regularly. 56 days after operation, animals were sacrificed for biomechanical and histological analysis. The national guidelines for the care and use of laboratory animals were observed and the study was approved by the Animal Experimental Ethics Committee of Berlin (approval number G0174/07).

### Radiographic evaluation

Radiographs were taken in lateral and posterior-anterior view at day 0 (operation) and at day 56 (sacrifice) with the use of digital X-ray cassettes (Fuji Photo Film Co., Fuji, Japan) and a Mobilett Plus X-ray unit (Siemens AG, Munich, Germany). The X-rays were evaluated by two observers.

### Biomechanical testing

All rats were sacrificed after 56 days and 10 animals of each group were taken for biomechanical analysis. The right femurs were dissected and soft tissue was removed. Afterwards, they were carefully prepared at the proximal and distal end to expose 3 mm of the implant on each side of the bone. The distal end was embedded with bone cement (Heraeus-Kulzer GmbH, Wehrheim, Germany) into a previously described push-out device [34], which was placed into a material testing machine (Zwick 1455, Ulm, Germany). During the test, the machine applied a constant linear propulsion (v = 2 mm/min) to the implant and the applied force was recorded. For measuring the bone-implant attachment strength, the peak force needed to loosen the implant was used. To minimize differences in the push-out force due to a different length of the bone or implant, maximum force was set in ratio to the total bone area in contact with the implanted K-wire [26,34]:

$$\begin{array}{c} \text{Strength}\;\text{of}\;\text{fixation}\;\sigma_{\text{u}}={\text{F}}_{\max}/\pi \text{DH}\;[\sigma_{\text{u}}:\text{strength}\;\text{of}\;\text{fixation}\;\left(\text{Mpa}\right),{\text{F}}_{\max}:\text{initial}\;\text{push-out}\;\text{force}\\ \left(\text{N}\right),\text{D}:\text{implant}\;\text{diameter}\left(\text{mm}\right),\text{H}:\text{Bone}\;\text{length}\;\left(\text{mm}\right)]. \end{array}$$

### Histomorphometry

**Figure 1:**
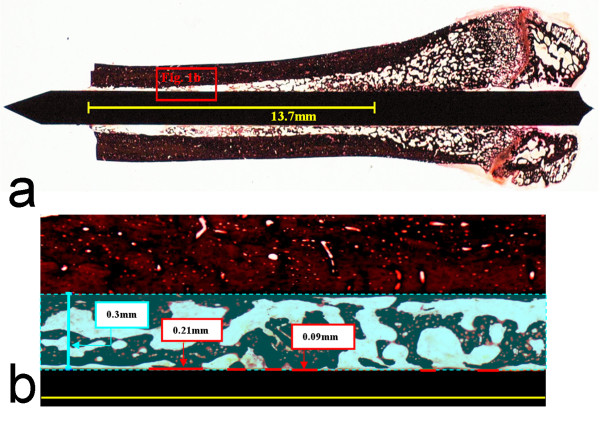
**(a) Histological sections of rat femurs (Region of interest: 13.7 mm from nutrient foramen; cutting zone of the bone) were analyzed (b) for bone/implant contacts and bone area (in a 0.3 mm spatium from implant)**.

For histological assessment the right femur of 10 animals of each group was harvested and cleared of soft tissue. The bones were fixed in 10% normal buffered formaldehyde for 5 days, dehydrated by ascending concentrations of ethanol and finally embedded in methylmethacrylate (Technovit 7200, Heraeus-Kulzer GmbH, Wehrheim, Germany). A grinding machine (Exakt, Norderstedt, Germany) was used to grind the embedded specimens until the implant was visible in full length with maximum diameter. After gluing the ground sides to microscope slides, a diamond band saw (Exakt, Norderstedt, Germany) was used to slice 300 μm sections which were ground down to 80 μm. The staining for histological analyses of the sections was performed with Safranin-O and von-Kossa. The specimens were scanned with a microscope (Axioskop 40, Carl Zeiss, Göttingen Germany). Direct bone/implant contact area was determined with analyzing software (AxioVision Rel. 4.7, Carl Zeiss AG, Jena, Germany). The region of interest (ROI) was defined by drawing a line of exact the same length (13.7 mm) in each digitalized picture, starting from the nutrient foramen (Figure 1a). Afterwards, bone/implant contact areas of both cortices were measured along the distance of the drawn line and set into per cent ratio. Furthermore, trabecular mass was measured within the ROI in an 0.3 mm range on both sides of the implant and set in per cent to the entire area of this spatium to determine bone area/total area ratio (Figure 1b).

### Statistics

Statistical differences between all groups were analyzed with the Kruskal-Wallis test. If significant differences were shown, Mann-Whitney U test was used for comparison between two single groups. Data were controlled with Bonferroni-Holm correction for multiple comparisons. Statistical differences were defined at a 95% confidence level. SPSS software (14.0; SPSS Inc., Chicago, IL) supported statistical evaluation.

## Results

### Release kinetics

**Figure 2:**
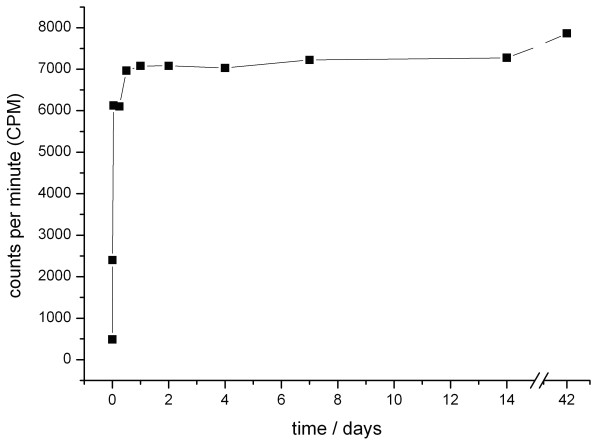
**In vitro elution kinetics of Carbon 14-labeled ZOL from a PDLLA coating with an initial burst release of ZOL within the first hours**.

The release of the incorporated C14-ZOL from the PDLLA coating showed a strong increase in detectable Counts Per Minute (CPM) within the first hours. In the following days and weeks, only a slight further progression of CPM took place, indicating that approximately 90% of C14-ZOL had been released in an initial peak within the first 24 hours (Figure 2).

### Body parameter evaluation and x-ray

No significant differences were detected in body temperature and raise of average body weight between the groups over the experimental period. None of the animals showed any clinical signs of infection throughout the experimental period.

**Figure 3:**
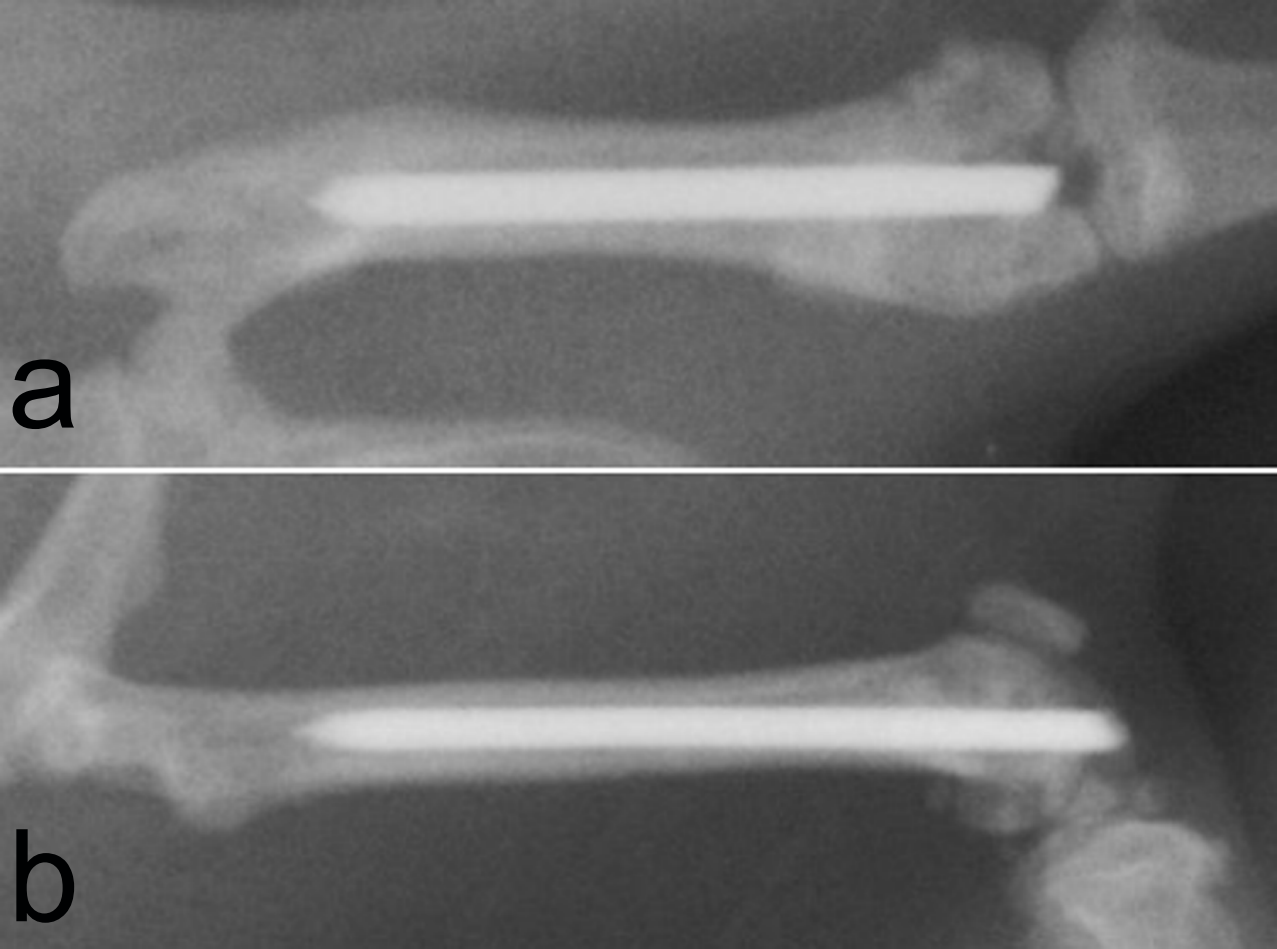
**X-rays (posterior-anterior (a) and lateral (b)) the operated leg of an animal of the PDLLA-treated group at time of sacrifice**.

Immediate postoperative X-rays revealed a correct insertion of each K-wire in all femurs. Evaluation criteria were here the placement of each implant inside the medullar canal with its tip positioned at the level of the lesser trochanter. The X-ray control after 56 days (Figure 3) showed no signs of dislocation of the implants or zones of osteolysis in all animals. Here, X-rays were analyzed in comparison to the postoperative records to evaluate if the medullar canal had been widened and if the position of the implants had varied, determined by the location of the K-wire tip and two points of contact with the cortical bone. Radiologically, there was no difference in implant/bone fitting between all groups.

### Biomechanical evaluation

**Figure 4:**
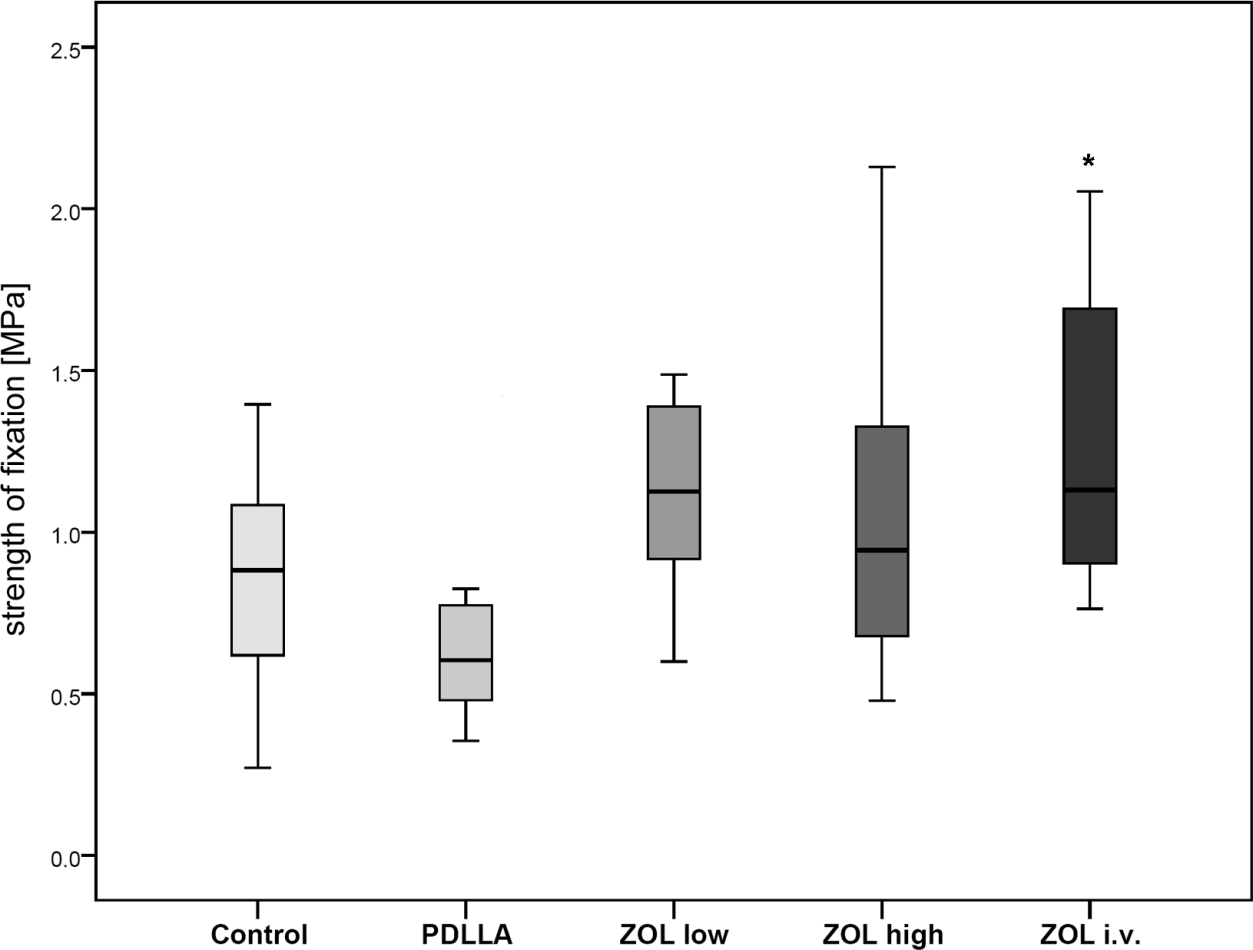
**Push-out strength of fixation (MPa)**. There were no significant differences between ZOL low/high and the other groups. However, the results of the ZOL i.v. group were significantly higher than those of the PDLLA group (* *p *= 0.002).

After determination of the strength of fixation by correlating the initial push-out force of each implant with the length of the surrounding bone, all groups were compared with each other. The results of the PDLLA group were slightly lower than those of the group with uncoated pins. No significant difference in strength of fixation was found comparing the ZOL low and ZOL high groups with the controls. The ZOL i.v. group showed no significant difference to the group with uncoated and ZOL-coated pins, whereas its results were significantly higher than those of the PDLLA group (Figure 4).

### Histomorphological and histomorphometric evaluation

**Figure 5:**
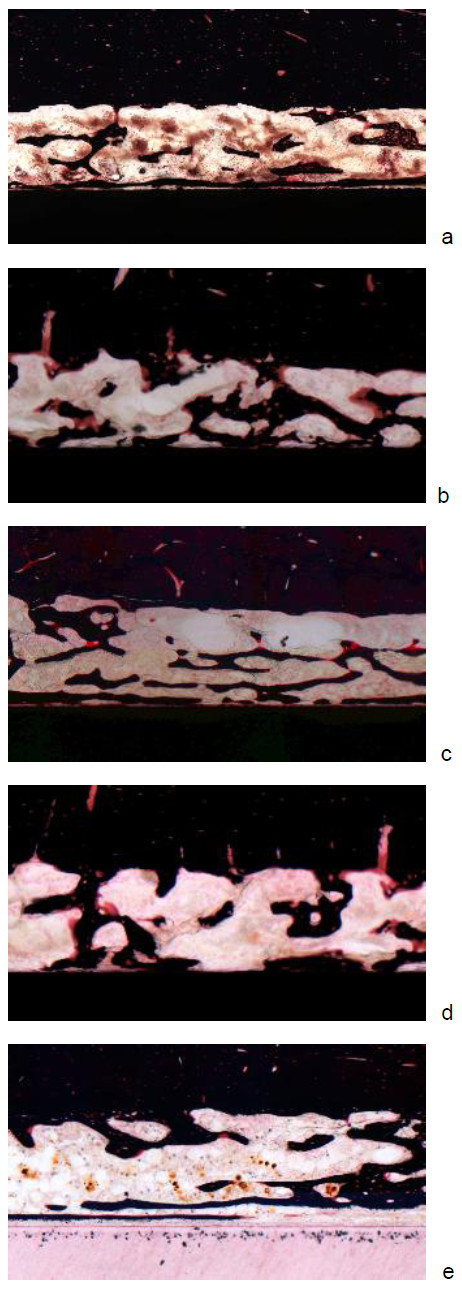
**a-Histological section of the femoral bone of an animal of the group without coating**. **b **- Histological sections of the femoral bone of an animal of the PDLLA-treated group. **c **- Histological sections of the femoral bone of an animal of the group with PDLLA/ZOL low coated implants. **d **- Histological sections of the femoral bone of an animal of the group with ZOL high coated implants. **e **- Histological sections of the femoral bone of an animal of the group which received ZOL intravenously.

**Figure 6:**
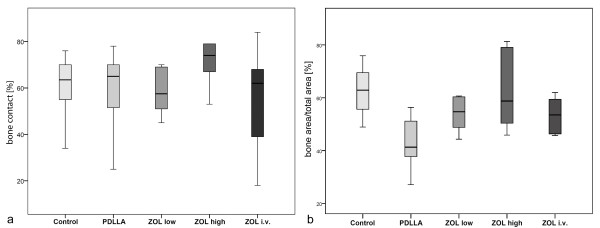
**a Bone/implant contact area (%)**. There were no significant differences between the investigated groups. **b **- Bone area/total area (%). There were no significant differences in bone area (%) in between the investigated groups.

Newly formed bone was visible in the femoral cavities of all animals, also in direct contact to the implants (Figure 5a-e). Regarding the amount of contact area of the newly formed intramedullar bone and the implant surface, there was no significant difference between all groups (Figure 6a). Also no significant changes in bone area/total area ratio were assessed between the groups (Figure 6b).

## Discussion

The purpose of this study was to investigate the release kinetics of Zoledronic acid (ZOL) incorporated in a poly(*D, L*-lactide) coating (PDLLA) and its effect on the osseointegration of implants, compared to a systemic ZOL application.

Release kinetics of ZOL out of the coating showed an initial peak of approximately 90% in release of C14-labled ZOL from PDLLA coated K-wires within the first 24 hours. The biomechanical and histological analyses revealed a significant higher strength of fixation in the group with systemic ZOL compared to PDLLA. However, no enhancement of osseointegration in groups treated with ZOL-coated implants in comparison to the controls could be shown.

These results are in contrast to other studies, showing an enhancement of osseo-integration or reduced implant migration after systemic [12,18,36] or local [1-3,8,19,27,28,30] application of Bisphosphonates (BPs). Especially the BP ZOL, as used in this study, has been shown to be among the most potent of its class [37]. However, existing studies vary in different aspects, such as additional use of bone compaction [2,3], the used BPs [7,27,28], the experimental model [14,20,30,38], animal species [2,7,30] implants [2,14,20,38] and application methods [7,38-40]. Among implant coatings, fibrinogen [4,5,38] and especially hydroxyapatite (HA) [20,27,30] have recently been used successfully to improve implant fixation by local application of BPs. Studies using ZOL locally were able to show an enhancement of peri-prosthetic bone quality and osseous integration by ZOL-coated implants with a similar animal model [20] or a significant enhancement of bone apposition and mean amount of femoral canal filling even after one year compared to controls [21]. Also a dose depending improvement of histological and biomechanical results by ZOL-application has been proved [14].

But there are also some studies where local BPs did not improve the bone-implant integration or even impaired it. After showing a benefit of local Alendronate in the fixation of porous-HA-coated implants [2] Jakobsen et al. detected a decrease in implant fixation when using soaked morselized allograft with the same dose and BP [29]. This effect, also shown in other studies with Pamidronate [28], could be due to the combination of densely compacted bone and BP [7]. But even in other study designs locally applied BPs did not always lead to significantly increased bone/implant contacts of BP-coated implants [22] nor did the additional application of BP enhance the biomechanical properties of coated implants [23].

Regarding systemic delivery, BPs have been shown to increase peri-implant bone density and implant-bone contact ratio in animal [39,40] and clinical [9] studies. Even though the same i.v. ZOL concentration (0.1 mg/kg) was used as by Yu et al. [39], no improvement in osseointegration was detected compared to the groups with PDLLA/ZOL or uncoated implants, except for a significant biomechanical enhancement in comparison to the PDLLA group. A possible reason could be the chosen time point of the application, being one week after surgery for Yu et al. vs. immediately during surgery in the presented study. This might have led to a stronger impairment of bone catabolism.

There are different explanations for the positive effects of BP in osseointegration. A reduction of implant migration by BPs, seen by Hilding and Aspenberg [19] was probably due to the inhibition of the resorption of periprosthetic necrotic bone [18].

Also bone formation around screws coated with fibrinogen, Pamidronate and Ibandronate was supposed to be based on reduced bone loss due to BP with the retained bone serving as scaffold for new bone cells [38]. Thus the enhancement of periprosthetic stabilization by BP appears to depend on the contact to surrounding bone and maybe even a press fit position [28,29]. Especially in a press fit situation the bone next to the implant may be necrotic and prone to resorption by osteoclasts. This resorption could be inhibited by BP, leading to an enhanced implant fixation.

In the present study, however, the implant fixation was not press fit in the medullary canal but in the cortex of the insertion point. Therefore neither local nor systemic inhibition of osteoclasts by ZOL might have supported additional implant ingrowth.

Since the time point of BP application seems to be decisive for its local effect on bone cells [39], data about **release kinetics **is important for a better prediction concerning the effect of the locally released BPs on the osseointegration of implants. Few other studies dealing with local release of BPs showed data for the individual specific **elution kinetics **[4,37]. Regarding the used coating, the present work is the first describing the **release kinetics **of ZOL out of the PDLLA coating [15,18-20].

However, even though the detected release kinetics with an initial peak confirms previous findings for the PDLLA coating [24] and though comparable kinetics between a phosphate buffered saline solution and cell culture medium have been shown [15], the here obtained releasing curve of ZOL cannot reflect the true release dynamics of intramedullary implants. Future studies will also have to investigate if the effect of the substance on bone cells could be improved by modification of the coating and variation of the release resulting in a slow sustained or delayed release [25]. In this context it could be tried to use C14-labled ZOL with autoradiographic analysis for local in vivo detection of released ZOL [16] as systemic detection would not be promising due to the high affinity of BPs to bones [13].

There are some limitations of this study. Among those should be seen the single time point (56 days), as no possible effect over time could be detected, even though other experiments have shown that osseous integration of implants was completed after six weeks [36]. Further limiting was the fact that the chosen implant model was not weight bearing and thereby effects of direct load transfer were not addressed.

Retrospectively, the use of a micro-CT with its possibility of a 3-D detection of newly formed bone or bone/implant contacts should be seen as best method for this purpose and would have avoided the danger of harming fragile structures like trabeculae by histological preparation. Another shortcoming was the lack of direct determination of the bioactivity of C14-ZOL after coating and release from PDLLA. However, previously published data has shown bioactivity of PDLLA-released ZOL on human bone cells [31-33].

## Conclusion

The time point of application as well as the way of implant fixation (press fit) seem to be decisive for the effect of BP in osseointegration. The presented model shows that a PDLLA/ZOL coating does not lead to an enhancement of osseointegration of non-press fit inserted implants. Further studies will be necessary to clarify if the mechanism of action of ZOL will lead to an improved osseointegration in a press fit implant fixation model and if a coating with a delayed release of the substance would lead to different findings in osseointegration.

However, in fracture fixation where strong bone/implant integration of intramedullary implants is an undesirable effect, local application of ZOL to stimulate fracture healing, as it has been described before [17], may still be an option. Since fracture stabilization devices are often explanted after consolidation, an enhanced osseointegration would pose an undesirable co-effect. Thus, the current findings are reassuring for further investigations of this coating in the context of fracture treatment with intramedullary implants.

## Competing interests

The authors declare that they have no competing interests.

## Authors' contributions

DB participated study design, coordination, in operations and drafted the manuscript. LR assisted in operations and performed the biomechanical and histological analyses. SG, SP, NH, GS and BW conceived the study, and participated in its design and coordination and helped to draft the manuscript. All authors read and approved the final manuscript.

## Pre-publication history

The pre-publication history for this paper can be accessed here:

http://www.biomedcentral.com/1471-2474/13/42/prepub
